# *D-aspartate oxidase* gene duplication induces social recognition memory deficit in mice and intellectual disabilities in humans

**DOI:** 10.1038/s41398-022-02088-5

**Published:** 2022-08-01

**Authors:** Barbara Lombardo, Marco Pagani, Arianna De Rosa, Marcella Nunziato, Sara Migliarini, Martina Garofalo, Marta Terrile, Valeria D’Argenio, Alberto Galbusera, Tommaso Nuzzo, Annaluisa Ranieri, Andrea Vitale, Eleonora Leggiero, Anna Di Maio, Noemi Barsotti, Ugo Borello, Francesco Napolitano, Alessandra Mandarino, Marco Carotenuto, Uriel Heresco-Levy, Massimo Pasqualetti, Paolo Malatesta, Alessandro Gozzi, Francesco Errico, Francesco Salvatore, Lucio Pastore, Alessandro Usiello

**Affiliations:** 1 CEINGE Biotecnologie Avanzate, 80145 Naples, Italy; 2 Dipartimento di Medicina Molecolare e Biotecnologie Mediche, Università di Napoli Federico II, 80131 Naples, Italy; 3 Functional Neuroimaging Laboratory, Center for Neuroscience and Cognitive Systems, Istituto Italiano di Tecnologia, 38068 Rovereto, Italy; 4 Unità di Biologia Cellulare e dello Sviluppo, Dipartimento di Biologia, Università di Pisa, 56126 Pisa, Italy; 5 Dipartimento di Scienze e Tecnologie Ambientali Biologiche e Farmaceutiche, Università degli Studi della Campania “Luigi Vanvitelli”, Caserta, Italy; 6 Dipartimento di Oncologia, Biologia e Genetica, Università di Genova, 16132 Genoa, Italy; 7 Dipartimento di Promozione delle Scienze Umane e della Qualità della Vita, Università San Raffaele, 00166 Rome, Italy; 8 Department of Veterinary Medicine and Animal Productions, University of Naples Federico II, 80137 Naples, Italy; 9 Clinic of Child and Adolescent Neuropsychiatry, Department of Mental Health, Physical and Preventive Medicine, University of Campania “Luigi Vanvitelli”, 80100 Naples, Italy; 10 Research and Psychiatry Departments, Ezrath Nashim-Herzog Memorial Hospital, 9190501 Jerusalem, Israel; 11 Hadassah Medical School, Hebrew University, 9190501 Jerusalem, Israel; 12 Dipartimento di Medicina Sperimentale, Università di Genova, 16132 Genoa, Italy; 13 Ospedale Policlinico San Martino IRCCS, 16132 Genoa, Italy; 14 Department of Agricultural Sciences, University of Naples Federico II, 80055 Portici, Italy; 15 Centro Interuniversitario per Malattie Multigeniche e Multifattoriali e loro modelli animali (Federico II, 80131, Naples; Tor Vergata, Rome and “G. D’Annunzio”, Chieti-Pescara), Naples, Italy; 16 Present Address: Novartis Ireland ltd, D04A9N6 Dublin 4, Ireland

**Keywords:** Neuroscience, Molecular neuroscience

## Abstract

The *D-aspartate oxidase* (*DDO*) gene encodes the enzyme responsible for the catabolism of D-aspartate, an atypical amino acid enriched in the mammalian brain and acting as an endogenous NMDA receptor agonist. Considering the key role of NMDA receptors in neurodevelopmental disorders, recent findings suggest a link between D-aspartate dysmetabolism and schizophrenia. To clarify the role of D-aspartate on brain development and functioning, we used a mouse model with constitutive *Ddo* overexpression and D-aspartate depletion. In these mice, we found reduced number of BrdU-positive dorsal pallium neurons during corticogenesis, and decreased cortical and striatal gray matter volume at adulthood. Brain abnormalities were associated with social recognition memory deficit at juvenile phase, suggesting that early D-aspartate occurrence influences neurodevelopmental related phenotypes. We corroborated this hypothesis by reporting the first clinical case of a young patient with severe intellectual disability, thought disorders and autism spectrum disorder symptomatology, harboring a duplication of a chromosome 6 region, including the entire *DDO* gene.

## Introduction

Glutamatergic signaling plays a pivotal role in brain development and functions as it regulates essential cellular events like proliferation, differentiation, and migration [1]. Consequently, alteration of this neurotransmission system has been associated with neurodevelopmental disorders such as schizophrenia (SCZ), autism spectrum disorder (ASD) and intellectual disability (ID) [2–4].

In addition to the NMDA receptor (NMDAR) co-agonist, D-serine [5–7], known to be involved in SCZ pathophysiology and treatment [8], another atypical D-amino acid, D-aspartate (D-Asp), has been shown to functionally modulate glutamatergic neurotransmission [9, 10]. In particular, D-Asp acts as an endogenous agonist of glutamatergic ionotropic NMDARs and metabotropic mGlu5 receptors (mGluR5) [11–14]. Noteworthy D-Asp is abundant in the prenatal brain of humans and rodents, while its levels are drastically reduced after birth [15–22]. The remarkable temporal regulation of cerebral D-Asp content is strictly controlled by postnatal expression onset of *D-aspartate oxidase* (*Ddo*) gene [21, 22], encoding the enzyme that catalyzes D-Asp degradation [23–25].

In line with its pharmacological features, elevated cerebral D-Asp content in adult mouse and rat models is associated with enhanced NMDAR-mediated synaptic plasticity [11, 26], as well as increased adult neurogenesis [27], dendritic length and spine density in pyramidal neurons of the prefrontal cortex (PFC) and hippocampus [26, 28], and cortical activity, as measured by functional magnetic resonance imaging (fMRI) [26, 28]. These results in rodents are consistent with observations in healthy humans showing that a *DDO* gene variant leading to reduced *DDO* expression is associated with greater prefrontal gray matter volume (GMV) and prefrontal activity, assessed during working memory tasks [26]. Conversely, reduced D-Asp levels and D-Asp/total Asp ratio, associated with either increased *DDO* mRNA expression or DDO enzymatic activity, have been reported in postmortem cortex from two distinct cohorts of SCZ patients [26, 29–31]. Coherently with findings in SCZ patients, D-Asp administration in preclinical models attenuated schizotypal symptomatology, such as prepulse inhibition (PPI) and brain activity dysfunction induced by the hallucinogenic drugs phencyclidine or MK801 [11]. Moreover, D-Asp metabolism alterations have been also recently found in the brain of an idiopathic animal model of ASD [32], thus further suggesting a possible involvement of D-Asp metabolism in regulating neurodevelopmental processes and related disorders.

In the attempt to decipher the unclear role of prenatal D-Asp metabolism in regulating the mammalian brain development, we used a knockin mouse model (*Rosa26*^*Ddo/+*^) mimicking a *Ddo* duplication in which this gene is constitutively overexpressed resulting in selective depletion of cerebral D-Asp levels from the very first developmental phases [22]. To address the consequences of D-Asp removal on brain development, we analyzed cortical neurogenesis through 5-bromo-2′-deoxyuridine (BrdU) immunohistochemistry and regional GMV through anatomical MRI. Moreover, we carried out comprehensive behavioral phenotyping to identify possible abnormalities relevant to neurodevelopmental disorders. Finally, during the screening by a-CGH methodology for search of large DNA alterations on genomic DNA in children and adolescents with ASD symptomatology, cognitive and behavioral retardation, we reported for the first time the identification of a patient with severe ID, thought disorders and other behavioral deficits carrying a DNA duplication region of approximately 127.8 kb in the 6q21 chromosomal region encompassing the entire *DDO* gene. Our data show that *Ddo* duplication in mice determines abnormalities in cortical development and social behavior defects, thus suggesting an influence of D-Asp metabolism in regulating neurodevelopmental processes that may contribute to the behavioral alterations observed in the patient with the *DDO* gene duplication.

## Methods

### Animals

All research involving animals was carried out in accordance with the directive of the Italian Ministry of Health governing animal welfare and protection (D.LGS 26/2014) and approved by “Direzione Generale della Sanità e dei Farmaci Veterinari (Ufficio 6)” (permission nr 796/2018). *Ddo* knockin mice were generated and genotyped by PCR as described previously [22]. All studies were performed on female wild-type *Rosa26*^*+/+*^ and heterozygous *Rosa26*^*Ddo/+*^ mice (*R26*^*+/+*^ and *R26*^*Ddo/+*^, respectively**)**, deriving from mating of *R26*^*+/+*^ females and *R26*^*Ddo/+*^ males, backcrossed into the C57BL/6J background for five generations (F5). This mating strategy was chosen to ensure preventing eventual effects of maternal D-Asp depletion on pups. To avoid potential complications in the interpretation of phenotypes due to hormonal fluctuations, we monitored the estrous cycle of both *R26*^*+/+*^ and *R26*^*Ddo/+*^ females [33] to ensure that they would always be tested at the same phase of the estrous cycle. Mice were housed in groups (*n* = 4–5) in standard cages (29 × 17.5 × 12.5 cm) at a constant temperature (22 ± 1 °C) and maintained on a 12 h light/dark cycle, with food and water ad libitum.

### In situ hybridization

In situ RNA hybridizations from brain and intestine tissues obtained, respectively, from postnatal day 0 (P0) and juvenile *R26*^*+/+*^ and *R26*^*Ddo/+*^ mice (*n* = 3/genotype) were performed as previously described [34] (see Supplementary Methods).

### Structural magnetic resonance imaging (MRI)

High-resolution MRI analysis of brains from adult *R26*^*+/+*^ (*n* = 9) and *R26*^*Ddo/+*^ (*n* = 8) mice was performed according to previously published protocols [35, 36]. Voxelwise intergroup differences in regional GM volume were mapped using a two-tailed Student’s *t* test (*t* > 2, *p* < 0.05) followed by an FWER cluster correction using a cluster threshold of *p* < 0.01 as implemented in FSL. Quantifications of GM volume were carried out in cubic region of interest (7 × 7 × 7 voxels isotropic) and intergroup comparisons of those quantifications in *R26*^*+/+*^ vs. *R26*^*Ddo/+*^ mice were carried out using a two-tailed Student’s *t* test (*t* > 2, *p* < 0.05). Further details are described in Supplementary Methods.

### BrdU incorporation and immunohistochemical analysis

For birth-dating experiments BrdU (Sigma, 100 μg/g body weight) was intraperitoneally injected twice (i.e., 9.00 a.m. and 3.00 p.m.) into timed *R26*^*+/+*^ pregnant females at E14.5 mated to *R26*^*Ddo/+*^ studs. Pups were killed at P0 (*n* = 4 *R26*^*+/+*^, *n* = 5 *R26*^*Ddo/+*^) and brains processed in agreement to a previous protocol [37]. BrdU immunohistochemistry experiments were analyzed by unpaired two-tailed Student’s *t* test. Further details are described in Supplementary Methods.

### Primary neuronal cultures and retroviral transduction procedures

Neural progenitor cultures were prepared from embryonic telencephalic explants as previously described [38]. Cells were plated at a density of 2.5 × 10^5^ cells/cm^2^ onto poly-D-Lysine coated coverslips and transduced by either *Ddo-EGFP* or *EGFP* control retroviral vectors immediately after plating and then grown for 7 days in SATO medium (see Supplementary Methods). Clonal analyses (*n* = 6; total number of clones Ctrl: 335, *Ddo*: 260) were evaluated with unpaired two-tailed Student’s *t* test.

### Mouse behavior

The whole behavioral characterization was carried out on juvenile/young adult (6–8 weeks) *R26*^*+/+*^ and *R26*^*Ddo/+*^ mice. Order of testing the animals was randomized and counterbalanced. Behavioral tasks were analyzed by blinded investigators.

#### Grooming and rearing

Habituated, individually housed animals (*n* = 8/genotype) were videotaped for 30 min under red light illumination according to a previous work [39] (see Supplementary Methods).

#### Novelty-induced exploration

Novelty-induced exploratory response was used to assess spontaneous motor function of mice (*n* = 15/genotype) according to a previous work [40] (see Supplementary Methods).

#### Accelerating rotarod test

The accelerating rotarod (Cat. No. 7650 UGO BASILE, Biological Research Apparatus, Varese, Italy) was used to test balance, motor coordination and learning of mice (*n* = 8/genotype) as previously described [40] (see Supplementary Methods).

#### Open field test

This paradigm was used to evaluate anxiety-like behavior and emotionality of mice (*n* = 8/genotype) [41] (see Supplementary Methods).

#### Elevated plus-maze test

This task was performed as described in the Supplementary Methods. Time spent by mice (*n* = 10/genotype) in the closed arms, center, and open arms (expressed in s) was analyzed.

#### Marble burying test

Mice (*n* = 10/genotype) were individually placed into a corner of a standard cage and left undisturbed for 30 min according to a previous report [42] (see Supplementary Methods).

#### Prepulse inhibition of the acoustic startle response

PPI of the acoustic startle response of mice (*n* = 8/genotype) was measured using the SR-Lab System (San Diego Instruments, San Diego, CA), and carried out according to a previous report [43] (see Supplementary Methods).

#### Social interaction

Social interaction test was performed in mice (*n* = 8/genotype) according to a previous work [44] (see Supplementary Methods).

#### Three-chamber sociability and social novelty test

The tasks were performed on mice (*n* = 14/genotype) according to a previous protocol [45] (see Supplementary Methods).

### Array CGH on humans

Written informed consent to perform genetic analysis was obtained from all subjects, according to the second Helsinki Declaration. High-resolution a-CGH analysis was performed on genomic DNA obtained from the peripheral venous blood of the patient at the age of 17 and her parents. DNA was extracted with the Illustra Nucleon Genomic DNA Extraction kit (GE Healthcare, UK), according to a previously described protocol [46–48] Further details are described in Supplementary Methods.

### Real-Time PCR (RT-PCR) analysis

RT-PCR analysis was performed as previously described [46]. A relative quantification using 9 genomic control DNA samples was carried out. The haploid gene copy number was calculated using the comparative delta-delta-*C*_*t*_ method. Further details are described in Supplementary Methods and Supplementary Table 2).

### *DDO* sequencing analyses in the human trio

We performed *DDO* direct sequencing on the whole subjects trio as previously reported [43] (see Supplementary Methods and Supplementary Table 3).

### Exome sequencing analysis

Exome sequencing was carried out in the family trio to identify possible additional genetic alterations using an NGS-based approach as previously specified [49, 50] (see Supplementary Methods).

### HPLC on human serum

Written informed consent to perform HPLC analysis was obtained from all subjects, according to the second Helsinki Declaration. Whole peripheral blood was drawn from the proband, and age- and gender-related control subjects (*n* = 7) by peripheral venipuncture according to a previously reported protocol [51]. Briefly, blood was collected into clot activator tubes (Kima, code 11020) and gently mixed. Samples were stored upright for 30 min at room temperature to allow blood to clot, and centrifuged at 2000 × *g* for 10 min at room temperature. Serum samples were then analyzed by HPLC as previously reported [32, 51–54] and described in Supplementary Methods. HPLC experiment was repeated and replicated three times.

### Statistical analyses

Sample size was determined on the basis of previous experiments and by using the software GPower. The normality distribution was tested using the Kolmogorov–Smirnov test. All data were expressed as mean ± standard error of measurement (SEM). Behavioral tasks were analyzed using unpaired two-tailed Student’s *t* test (grooming, rearing, elevated plus maze, marble burying test, social interaction, analysis of discrimination index in three-chamber sociability and social novelty tasks), two-way ANOVA (analysis of time spent sniffing in the three-chamber sociability and social novelty tasks) followed by Fisher’s post hoc comparison, or two-way ANOVA with repeated measures (novelty-induced exploration, rotarod, open field, PPI). MRI, BrdU immunohistochemistry and clonal size data were analyzed by two-tailed Student’s *t* test. No samples or animals were excluded from the analyses.

## Results

### *Ddo* gene duplication affects neuronal proliferation and cortico-striatal gray matter volume in mice

**Fig. 1:**
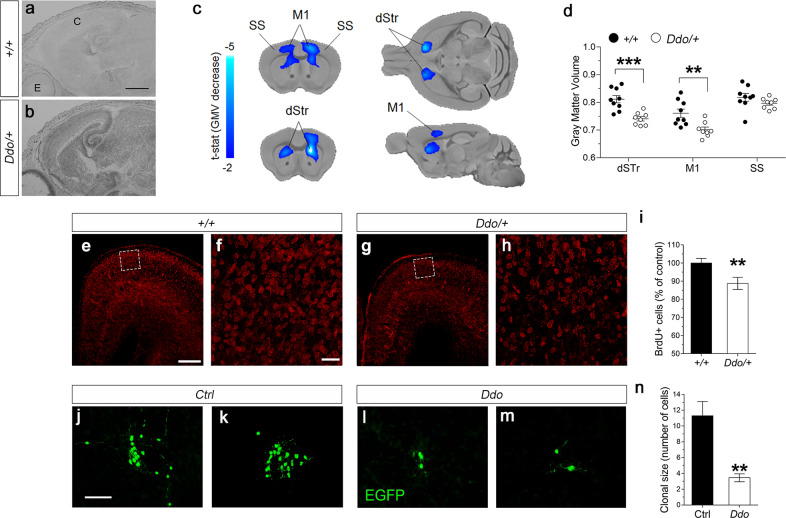
Reduced gray matter volume in motor cortex and striatal regions of adult Ddo knockin mice and lower number of cortical neurons in the dorsal pallium of newborn pups. **a**, **b** Representative images of sagittal sections showing *Ddo* expression in (**a**) *R26*^*+/+*^ and (**b**) *R26*^*Ddo/+*^ pups at birth. C cortex, E eye. Scale bar 1 mm. **c** High-resolution structural MRI revealed a prominent reduction of gray matter volume (GMV) in cortical and striatal areas of *R26*^*Ddo/+*^ compared to *R26*^*+/+*^ mice. **d** Regional volumetric analysis of GMV in *R26*^*+/+*^ and *R26*^*Ddo/+*^ mice in dorsal striatum (dStr), primary motor cortex (M1) and somatosensory cortex (SS). **e–i** Birth-dating experiment of embryos injected with BrdU at E14.5. **e–h** Representative coronal sections showing BrdU-positive neurons in (**e**, **f**) *R26*^*+/+*^ and (**g**, **h**) *R26*^*Ddo/+*^ mice at birth. **f**, **h** Confocal higher magnification of the boxed region in the dorsal pallium. Images further demonstrate the reduced density of BrdU-positive cells in (**h**) *R26*^*Ddo/+*^ pups as compared to (**f**) *R26*^*+/+*^ controls. Scale bars: (**e**, **g**) 250 μm, (**f**, **h**) 50 μm. **i** Bar graph showing the reduction of the BrdU-positive cells in the dorsal pallium of *R26*^*Ddo/+*^ pups. **j**, **m** Representative images of clones generated by single E14 telencephalic progenitor cells transduced with retroviral vectors. Scale bar 100 µm. **n** Bar graph showing the average size of clones generated by the transduced progenitor cells (*n* = 6, total number of clones, Ctrl: 335, *Ddo*: 260). All data are expressed as mean ± SEM. **p < 0.01; ***p < 0.0001, compared with control group (Student’s *t* test).

We used the recently generated *R26*^*Ddo/+*^ mouse model in which the exogenous *Ddo* gene is constitutively overexpressed resulting in selective depletion of cerebral D-Asp content starting from prenatal life [22]. In line with quantitative RT-PCR analysis [22], in situ hybridization revealed increased *Ddo* mRNA expression throughout the brain of P0 *R26*^*Ddo/+*^ mice, as compared to age-matched *R26*^*+/+*^ littermates (Fig. 1a, b), which correlates with the depletion of cerebral D-Asp levels in this mutant mouse model [22]. We also evaluated peripheral D-Asp occurrence in the blood serum and intestine of juvenile *R26*^*Ddo/+*^ mice. Statistical analysis in the mouse serum revealed decreased D-Asp concentration and D-Asp/total Asp (D + L) ratio in *R26*^*Ddo/+*^ mice, compared to *R26*^*+/+*^ littermates (D-Asp, *p* = 0.0412; L-Asp, *p* = 0.2482; D-Asp/total Asp, *p* < 0.0001; Student’s *t* test; Supplementary Fig. 1a–d). Also in the intestine we observed D-Asp and D-Asp/total Asp ratio reduction in *R26*^*Ddo/+*^ mice compared to control littermates **(**D-Asp, *p* = 0.0031; L-Asp, *p* = 0.1813; D-Asp/total Asp, *p* = 0.0008, Student’s *t* test; Supplementary Fig. 2h–j). Conversely, in this organ we did not observe any alteration in the amount of other amino acids, including D-serine, L-serine, L-glutamate, L-glutamine, L-asparagine and glycine (Supplementary Fig. 2k–q). In line with the decrease of intestinal D-Asp levels, in situ hybridization and quantitative RT-PCR analysis revealed increased *Ddo* mRNA expression in the intestine of juvenile *R26*^*Ddo/+*^ mice, as compared to age-matched *R26*^*+/+*^ mice (RT-PCR: *Ddo*, *p* = 0.0006; Supplementary Fig. 2a–e). Conversely, no differences were found in the expression levels of *D-amino acid oxidase* (*Daao*) and *Serine racemase* (*Sr*), which are the genes responsible for D-Ser metabolism [55, 56] (Supplementary Fig. 2f, g). The latter gene has been suggested to participate also to D-Asp biosynthesis in some brain regions [57, 58]. To investigate whether *Ddo*-overexpressing mice exhibit brain-wide neuroanatomical alterations, we employed high-resolution structural MRI to obtain spatially unbiased maps of GMV with voxel resolution [35]. Intergroup genotype-dependent comparisons revealed prominent bilateral reductions of GMV in the motor cortex of adult *R26*^*Ddo/+*^ mice, compared to control littermates (*t* test, *t* > 2, *p* < 0.05, FWER cluster-corrected defining threshold of *p* < 0.05; Fig. 1c). In keeping with this finding, volumetric mapping revealed also a focal bilateral loss of striatal GMV in *R26*^*Ddo/+*^ mice as compared to control mice (*t* test, *t* > 2, *p* < 0.05, FWER cluster-corrected defining threshold of *p* < 0.05, Fig. 1c). Regional quantifications of GM in regions of interest confirmed the volumetric loss in the motor cortex (*t* = 3.227, *p* = 0.006) and striatal areas (*t* = 4.427, *p* = 0.0005, Fig. 1d). Quantifications of total brain volume revealed unimpaired intracranial volume in *R26*^*Ddo/+*^, compared to control animals (*t* test, *t* = 0.79, *p* = 0.44, data not shown).

Stimulation of NMDARs and mGluR5 subtypes is reported to induce neuronal proliferation and cell migration [1]. Accordingly, we investigated the influence of D-Asp metabolism on cortical neurogenesis both in vivo and in vitro. To determine the origin of the reduced cortical volume observed by MRI in adult *Ddo*-overexpressing mice, we analyzed the generation of cortical neurons in the dorsal pallium, the prospective structure of the brain region where the cortical phenotype was observed. To this aim, we performed a birth-dating experiment injecting the thymidine analog BrdU at E14.5 to label cycling cortical progenitor cells. Interestingly, a decrease in BrdU-positive cells was observed at P0 (−12%, *t* = 2.77 *p* = 0.0078; unpaired two-tailed Student’s *t* test; Fig. 1e–i), demonstrating a reduced neurogenesis in the developing cortex of animals with additional *Ddo* gene copy.

To explain these in vivo findings and directly link the neuronal proliferation phenotype to DDO misexpression, we overexpressed DDO enzyme in E14 telencephalic progenitor cells via retroviral transduction. Remarkably, clonal analysis of neural progenitor cultures showed a significant reduction of the average size of clones after 1 week of in vitro growth (*p* < 0.01, Student’s *t* test; Fig. 1j–n). These findings confirmed a *Ddo*-dependent reduction in neuronal cell proliferation, which may be related to D-Asp levels regulation.

### *Ddo*-overexpressing mice exhibit selective social recognition deficit

Consistent with a key role of NMDAR and mGluR5 signaling in modulating a wide range of behaviors relevant to neuropsychiatric disorders, here we explored whether the congenital depletion of D-Asp could affect some of these in vivo phenotypes at juvenile/young adult phase (6–8 weeks), including stereotypic behavior, motor activity, motor learning and coordination, sensorimotor gating, anxiety-related responses, compulsivity and social behavior.

**Fig. 2:**
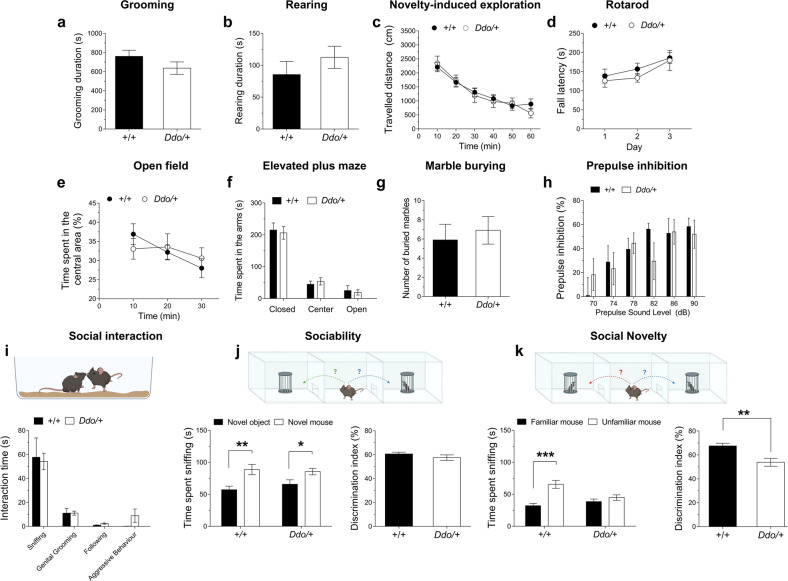
Ddo-overexpressing mice exhibit selective social recognition deficit. **a–k** *R26*^*Ddo/+*^ and *R26*^*+/+*^ mice were tested in a series of behavioral tasks relevant to psychiatric phenotypes. **a**, **b** Analysis of grooming and rearing (duration, expressed in s; *n* = 8/genotype), (**c**) novelty-induced exploration test (traveled distance; expressed in cm, *n* = 15/genotype), (**d**) rotarod test (fall latency, expressed in s; *n* = 8/genotype), (**e**) open field test [time spent in the central area, expressed as percentage (%) of total time (peripheral + central area); *n* = 8/genotype], (**f**) elevated plus-maze test (time spent in the arms, expressed in s; *n* = 10/genotype), (**g**) marble burying test (expressed as number of buried murbles; n = 10/genotype), (**h**) prepulse inhibition of the startle reflex [expressed as percentage (%); *n* = 8/genotype], (**i**) social interaction (interaction time, expressed in s; *n* = 8/genotype), (**j**) sociability and (**k**) social novelty test (*n* = 14/genotype). **j**, **k** Sociability and social novelty tests were analyzed as both time spent sniffing (expressed as s, left graphs) and discrimination index [expressed as the ratio between the time spent exploring the novel stimulus and the total exploration time, according to the following formulas: (i) sociability test: (time spent with mouse)/(time spent with mouse + time spent with object); (ii) social novelty test: (time spent with novel mouse)/(time spent with novel mouse + time spent with familiar mouse), right graphs]. **i–k** Representative drawings of the social behavior tasks are shown above graphs. All data are expressed as mean ± SEM. *p < 0.05; **p < 0.01, compared with novel object; ***p < 0.0001, compared with familiar mouse (Fisher’s post hoc comparison).

First, we tested stereotypies, such as grooming and rearing, in *R26*^*Ddo/+*^ mice and control littermates. We found that D-Asp depletion did not impact on either grooming (*p* = 0.1988, Student’s *t* test) or rearing (*p* = 0.3334, Student’s *t* test) activity of *R26*^*Ddo/+*^ mice, compared with *R26*^*+/+*^ controls (Fig. 2a, b). Then we analyzed spontaneous locomotor activity by novelty-induced exploration test [59]. We observed a comparable habituative profile of locomotion between genotypes (two-way ANOVA, genotype: F_(1,28)_ = 0.04047, *p* = 0.8420; time: *F*_(5,140)_ = 45.19, *p* < 0.0001; genotype × time: *F*_(5,140)_ = 1.002, *p* = 0.4188; Fig. 2c). We also evaluated motor coordination and motor learning by accelerating rotarod test [40]. Data showed that *R26*^*Ddo/+*^ and *R26*^*+/+*^ mice had similar latency to fall during a 3-day training trial (two-way ANOVA, genotype: *F*_(1,14)_ = 0.4853, *p* = 0.4974; days: *F*_(2,28)_ = 8.046, *p* = 0.0017; genotype × days: *F*_(2,28)_ = 0.2211, *p* = 0.8030, Fig. 2d). To explore the impact of early D-Asp deprivation on anxiety, we performed open field and elevated plus-maze tasks [60]. In the open field test, animals of both genotypes spent comparable amount of time in the center region of the arena (two-way ANOVA, genotype, *F*_(1,14)_ = 0.0002975, *p* = 0.9865; genotype × time *F*_(2,28)_ = 1.922, *p* = 0.1651, Fig. 2e). In the elevated plus-maze test, *R26*^*Ddo/+*^ and *R26*^*+/+*^ mice tended to stay both in the closed arms of the apparatus (*p* = 0.7678, Student’s *t* test, Fig. 2f). Next, we assessed compulsivity by analyzing repetitive digging behavior using the marble burying test [42]. Also in this case, we failed to observe any difference between genotypes in the number of marbles buried (*p* = 0.6512, Student’s *t* test, Fig. 2g). Similarly, analysis of sensorimotor gating through PPI of the startle reflex did not reveal significant alterations in *R26*^*Ddo/+*^ mice, compared with *R26*^*+/+*^ animals (two-way ANOVA; genotype, *F*_(1,14)_ = 0.04656, *p* = 0.8323; decibel levels, *F*_(5,70)_ = 7.56, *p* < 0.0001; genotype × decibel levels, *F*_(5,70)_ = 1.339, *p* = 0.2580, Fig. 2h).

Finally, we evaluated social behavior. We first performed social interaction test to evaluate spontaneous parameters of mouse sociability, like sniffing, genital grooming, following, aggressivity [44]. None of these behaviors was affected in *R26*^*Ddo/+*^ mice, compared to *R26*^*+/+*^ controls (sniffing: *p* = 0.8441; genital grooming: *p* > 0.9999; following: *p* = 0.0668; aggressive behavior: *p* = 0.1364; Student’s *t* test, Fig. 2i). Next, we performed three-chamber sociability and social novelty test [45]. In the sociability test, no significant difference between genotypes was found in terms of social investigation (two-way ANOVA, genotype, *F*_(1,52)_ = 0.1737, *p* = 0.6786; sniffing time, *F*_(1,52)_ = 15.41, *p* = 0.0003; genotype × sniffing time, *F*_(1,52)_ = 0.8086, *p* = 0.3727, Fig. 2j). Indeed, both *R26*^*Ddo/+*^ and *R26*^*+/+*^ mice spent more time sniffing the cage containing the novel mouse compared to the empty cage (*R26*^*+/+*^, *p* = 0.0013; *R26*^*Ddo/+*^, *p* = 0.0370; Fisher’s post hoc comparison, Fig. 2j, left graph). In a subsequent test phase, a novel social partner (unfamiliar) was introduced into the previously empty cage to evaluate social novelty. Remarkably, we found a significant difference in preference for social novelty between genotypes (two-way ANOVA, genotype, *F*_(1,52)_ = 2.368, *p* = 0.1299; sniffing time, *F*_(1,52)_ = 18.17, *p* < 0.0001; genotype × sniffing time, *F*_(1,52)_ = 8.549, *p* = 0.0051, Fig. 2k). Indeed, *R26*^*+/+*^ mice displayed a natural preference for the novel animal, as shown by the increase in time spent sniffing the unfamiliar mouse, compared with the familiar one (*p* < 0.0001, Fisher’s post hoc comparison, Fig. 2k, left graph), while *R26*^*Ddo/+*^ mice spent comparable time interacting with familiar and unfamiliar mice (*p* = 0.3482, Fisher’s post hoc comparison, Fig. 2k, left graph). Such specific impairment in *R26*^*Ddo/+*^ mice was evident also when we reported data as discrimination index that measures the ratio between the time spent exploring the novel stimulus and the total exploration time (*R26*^*+/+*^ vs. *R26*^*Ddo/+*^: sociability, *p* = 0.0022; social novelty, *p* = 0.2673; Student’s *t* test; Fig. 2j, k, right graphs).

Taken together our data showed that constitutive lack of cerebral D-Asp since prenatal life produces a selective social novelty deficit in juvenile *Ddo*-overexpressing mice.

### Clinical abnormalities in a patient with *DDO* gene duplication

While enrolling by high-resolution a-CGH analysis children and adolescents (up to 20 years old) with ASD symptomatology, cognitive and behavioral retardation to search for large DNA alterations on genomic DNA, we obtained in a patient of 17 years old (J.R.) a DNA duplication of 127.8 kb containing the entire *DDO* gene, in heterozygosity. J.R. referred to our Hospital (Clinic of Child and Adolescent Neuropsychiatry, University of Campania “Luigi Vanvitelli”, Italy) for her behavioral problems and consequent psychopharmacological therapy.

J.R. was born at 41 weeks after a normal pregnancy with a pharmacologically-induced delivery by non-consanguineous parents; she had a family history of cardiopathy in the paternal line. At the first examination, the newborn showed a normal birthweight (3300 g) and good adaptation to extra-uterine life. At 2 years of age, delays in autonomous gait, motor clumsiness and speech were detected. At 3 years of age, the first neurological evaluation showed difficulties in motor coordination, dissymmetry, motor stereotypies and language impairment. Consequently, a psychomotor developmental delay was diagnosed and, since then, she was periodically evaluated and treated with rehabilitation therapies. The patient was admitted at nursery school at 2 years of age and demonstrated good socialization skills. Subsequently, she has always needed support for school and personal care. She needed speech and neurocognitive support and was treated with cognitive-behavioral therapy, occupational and physical therapy.

She had menarche at about 10 years of age, with irregular cycles always characterized by menorrhagia and primary dysmenorrhea. The family rejected the recommended hormone therapy for possible effects on her weight. At the age of 11, standard karyotype, high-resolution karyotype and molecular analysis for Prader Willi syndrome, Angelman syndrome and fragile-X syndrome were performed and they all resulted negative. Additional laboratory tests, including acid-base equilibrium (ABE), aminoaciduria and urinary organic acids, did not indicate specific pathologies. In addition, a full cardiological assessment, brain MRI and fundus oculi analysis indicated absence of pathological signs. Electroencephalogram (EEG) showed non-specific alterations with presence of a moderate and diffuse slowed background activity.

The physical examination at age 17 revealed several dysmorphisms such as low hairline, hypertrichosis, micrognathia, simplified ear cupped, microcephaly (below −2 DS) and height at 10th centile. At the same age, neurological evaluation revealed good visual pursuit, exotropia of the left eye, nystagmus in lateral view, motor clumsiness, oscillating gait with enlarged base, sporadic toe walking, ligamentous laxity. Tendon reflexes were present. A number of hand stereotypes, deficit of hand-eye coordination, both in motor-praxis abilities and in fine motor skills were observed.

At age of 17, J.R. was attending high school with providing educational support including a reduced school hours program and specific after-school activities. She seemed very sociable and inclined to relational exchanges. The language was poorly structured and characterized by echolalia and soliloquy. Her thought was frequently tangential and incongruous; there was no evidence of dysperceptions. The emotional expressiveness was often inadequate with a dysphoric characterization. When her routine was changed, she had uncontrolled reactions, characterized mainly by self-aggressive behaviors. She also showed severe impulsivity, psychomotor agitation, frustration intolerance, and a severe deficit in attention span. In contrast, good short- and long-term memory was observed. A neuropsychological assessment performed using the Wechsler scale (WISC III) evidenced a severe ID (<40 vs. 85–115 normal values). The Raven’s Matrices test was not performed due to patient’s lack of compliance. Her adaptive profile, as detected by Vineland Adaptive Behavior Scales (VABS) questionnaire, was below normal levels (<40 vs. 85–115 normal values).

### Identification of *DDO* duplication

**Fig. 3:**
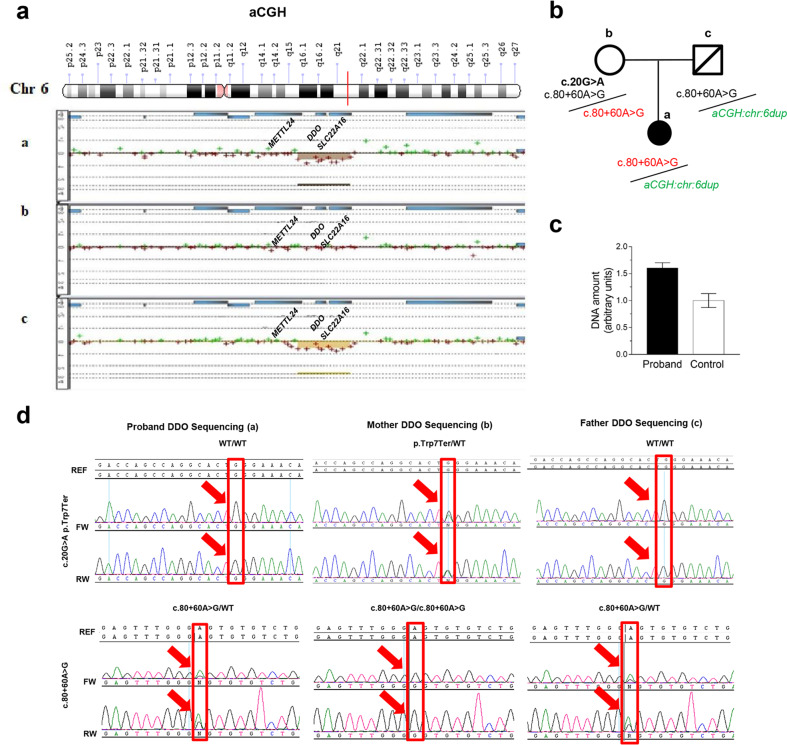
Identification of DDO gene duplication and single nucleotide variants. **a** a-CGH profile of chromosome 6 from the Agilent 4X180K array revealed duplication in 6q21 of ~128 kb involving *DDO*. Results are interpreted as log2 ratio of test versus control (in case of no mutation log2(2/2) = 0, in case of duplication: log2(3/2) > 0.58). The patient (**a**) exhibits a duplication inherited from her father (deceased) (**c**), absent in the mother (**b**). The green and red dots represent the log2 fluorescence ratios of individual oligonucleotide probes on the microarrays. The red dots are indicative of the duplication segment on chromosome 6 and represent probes with positive log2 fluorescence ratios; the green dots represent probes with negative log2 fluorescence ratios. **b** Family trio pedigree reporting the DNA sequence variants identified in each family member by *DDO* sequencing analysis. The only variant found in the proband (c.80-60A>G) (**a**) is inherited by the mother (**b**). The mother (**b**) of the patient was found to carry a nonsense mutation (c.20G>A; p.Trp7Ter) not previously reported. **c** RT-PCR on proband for *DDO* gene. **d** *DDO* gene Sanger Sequencing electropherograms: the proband (**a**) is wild-type for the nonsense variant c.20G>A (p.Trp7Ter) as the father (**c**), the mother shows the variant in heterozygosity. The proband presents the variant c.80 + 60A>G in herozygosity as the father, while the mother is homozygous for the same variant. F forward strand, R reverse strand, REF reference sequence. The red large arrows and the rectangles indicate the exact position of each variant at sequence level.

a-CGH analysis was carried out on the proband and her parents to assess the presence of chromosomal alterations which could be possibly related to the patient’s clinical phenotype. We identified in the proband a duplication of ~127.8 kb in the chromosome 6 at q21 region described as: chr6.hg19:g.(110,668,913 _110,668,972)_(110,796,717_110,796,764)dup (Fig. 3a and Supplementary Fig. 3). The duplication was paternally inherited and located between array clones A_16_P37749162 and A_14_P100519 (Fig. 3a, b). We confirmed the presence of this duplication in the patient by quantitative RT-PCR (Fig. 3c).

As evaluated using the Database of Genomic Variants, the DECIPHER Database and the UCSC Genome Browser, the duplication observed in the patient includes the entire *DDO* gene (RefSeq # NC_000006) and part of the *methyltransferase-like 4* (*METTL24*) gene at the 5′ end of the duplicated DNA segment, and *Solute Carrier Family 22 Member 16* (*SLC22A16*) gene at the 3′ end of the same duplication (Fig. 3a and Supplementary Fig. 3).

### *DDO* gene and exome sequencing analysis

Sequence analysis of the *DDO* gene performed by Sanger Sequencing (SS) methodology (Table 1a) was carried out in the family trio to analyze the presence of single nucleotide variants and/or small insertions/deletions, independently inherited by the proband. We found that the patient carries just one common heterozygous deep-intronic variant in *DDO* intron 1, c.80 + 60A>G, (Fig. 3d and Table 1a). In addition, the patient’s mother was found to carry a nonsense variant [c.20G>A; p.(Trp7Ter)] in heterozygous status, which was not previously reported (Fig. 3b, d and Table 1a).

**Table 1:** **a–c. a** DNA sequence variants identified in each family member in the *DDO* gene (ENST00000368924.7) using direct sequencing (Sanger Sequencing). **b** List of variants found in the duplicated region (chr:6,110668913-110796764) besides *DDO* gene of the patient by exome sequencing. **c** List of pathogenic variants found in the exome analysis besides *DDO* gene in the proband.

**a**									
**Patients**	**cDNA**	**Protein**	**Hom/Het**	**Codons**	**Reference SNP ID (rs)**	**ClinVar database**	**ACMG Classification**^**a**^	**gnomAD frequencies**^**b**^	**Pathogenic Predictions (*n*°)**^c^
Proband	c.80 + 60A>G	–	Het	–	rs9384742	Not reported	Benign	*ƒ* = 0.657	–
Mother	c.20G>A	p.Trp7Ter	Het	tGg/tAg	rs141023778	Not reported	Uncertain Significance	*ƒ* = 0.00142	Positive in: 3 out of 9
	c.80 + 60A>G	–	Hom	–	rs9384742	Not reported	Benign	*ƒ* = 0.657	–
	c.543-39T>A	–	Het	–	rs2235989	Not reported	Benign		–
Father	c.80 + 60A>G	–	Het	–	rs9384742	Not reported	Benign	*ƒ* = 0.657	–

**b**							
**Gene**	**Transcript**	**cDNA change**	**Amino acid change**	**Reference SNP ID (rs)**	**Hom/Het**	**Disease related**	**Inheritance**
*METTL24*	NM_001123364.2	c.417 + 21 A>G	–	rs6908306	Het	–	–
		c.318 + 96A>C	–	rs4945854	Hom		
		c.63T>C	p.Ala21=	rs62435951	Hom		
*SLC22A16*	NM_033125.3	c.1522-238A>G		rs7756222	Het	Clear cell adenocarcinoma, rhizomelic chondrodysplasia punctata, ovarian cancer	AR
		c.1521 + 85C>T	–	rs1033572	Het		
		c.312T>C	–	rs6907567	Het		
		c.146A>G	p.Asn104=	rs714368	Het		
		c.53 + 1678A>T	p.His49Arg	rs7767808	Het		
		c.53 + 1603T>A	–	rs7747628	Het		
		c.53 + 756A>C	–	rs55679760	Hom		
		c.53 + 20C>G	–	rs62421706	Hom		
		c.53 + 18C>G	–	rs62421707	Hom		
		c.53 + 17C>T	–	rs62421708	Hom		
		c.53 + 16C>G	–	rs62421709	Hom		
		c.53 + 15C>G	–	rs62421710	Hom		
		c.−44T>C	–	rs11153236	Hom		
**c**							
*SLC6A19*	NM_001003841.2	c.1017-4G>A	–	rs35329108	Hom	Iminoglycinuria, Hyperglycinuria	AR
*PRKCQ*	NM_001323265.1	c.989C>T	p.Pro330Leu	rs2236379	Het	Inflammatory bowel disease	Mu
*UCP3*	NM_003356.3	c.304G>A	p.Val102Ile	rs2229707	Het	Obesity and Diabetes Type II	AD, AR, Mu
*TYR*	NM_000372.4	c.1037-7T>A	–	rs61754381	Het	Oculocutaneous albinism type 1B	AR
*ERCC4*	NM_005236.2	c.1730dupA	p.Tyr577Ter	rs397509404	Het	Cockayne Syndrome	AR
*SLC12A3*	NM_000339.2	c.1946C>T	p.Thr649Met	rs145337602	Het	Familial hypokalemia-hypomagnesemia	AR
*SERPINA7*	NM_000354.5	c.909G>T	p.Leu303Phe	rs1804495	Het	Thyroxine-binding globulin QTL	XL

*Hom* Homozygous, *Het* Heterozygous, *SNP* Single Nucleotide Polymorphism, *AR* Autosomal Recessive Disease, *AD* Autosomal Dominant Disease, *Mu* Multifactorial disorder, *XL* X-linked Disease.

*METTL24*: chr6:110,567,131-110,679,475; *SLC22A16*: chr6:110,745,890-110,797,844.

^a^ACMG: American College of Medical Genetics and Genomics (Richards S., et al. Standards and guidelines for the interpretation of sequence variants: a joint consensus recommendation of the American College of Medical Genetics and Genomics and the Association for Molecular Pathology. Genet Med. 2015;17:405–24).

^b^GnomAD: The Genome Aggregation Database is a resource that aggregates exome and genome sequencing data. The v2 data set (GRCh37/hg19) spans 125,748 exome sequences and 15,708 whole-genome sequences from unrelated individuals (Data released on October 2018).

^c^These are the 9 tools used for bioinformatic prediction by VarSome site. (1) BayesDel addAF, (2) BayesDel noAF; (3) MutationTaster, (4) EIGEN; (5) EIGEN PC; (6) FATHMM-MKL; (7) FATHMM-XF; (8) LRT; (9) ALoFT. For the first 3 tools the variant c.20G>A (p.Trp7Ter) is Pathogenic/Damaging, for the others it shows as Benign/Neutral effect.

To exclude the presence of independently inherited single nucleotide variants in genes other than *DDO*, we analyzed the whole trio by clinical exome sequencing. Exome sequencing resulted in a number of reads ranging between 63,741,159 and 65,478,691. The percentage of reads that passed the quality filters was up to 96% for all three subjects and the average read depth in the analyzable target regions was up to 85X. We found more than 73,000 variants/subject (Supplementary Table 1): variants’ distribution showed that about 26,000 variants were in the exons, more than 32,000 in the introns, 11,000 in intergenic regions and more than 4,000 in upstream and downstream regions (Supplementary Fig. 4). In Table 1b, after exome sequencing, we documented *METTL24* and *SLC22A16* gene variants belonging to the large duplication region surrounding *DDO* gene; no significant alterations could be evidenced.

Moreover, outside the duplicated DNA region, the exome sequencing revealed seven different pathogenic/likely pathogenic variants in the proband genome according to ClinVar Database (Table 1c). Six of the seven variants were in heterozygous state and associated with autosomal recessive diseases. One of them, the c.1017-4G>A—rs35329108 in *SLC6A19* gene, was in homozygous state in the proband. This variant is now classified as benign both in ClinVar Database and also for ACMG classification, although in some papers it was reported that it may contribute to iminoglycinuria/hyperglycinuria (disorders of renal tubular transport affecting reabsorption of glycine, proline and hydroxyproline) when combined with mutations in genes encoding other SLC amino acid transporters [61].

### Proband D-aspartate and L-aspartate serum concentration

To investigate the biochemical consequences of *DDO* gene duplication on D-Asp metabolism, we measured D-Asp and L-Asp levels by HPLC in the patient’s serum (at the age of 20), and compared the values with those of age- and sex-matched control subjects.

**Fig. 4:**
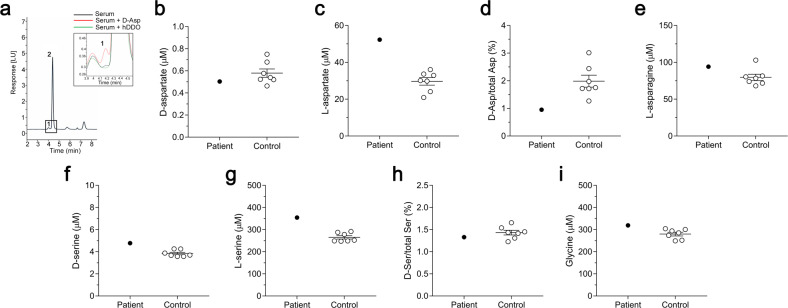
Determination of D-aspartate, L-aspartate and other amino acids content in blood serum sample of the patient. **a** Overlaid HPLC chromatograms illustrating the specificity of D-aspartate (D-Asp) peaks obtained from blood serum samples. The identity of the peak corresponding to D-aspartate was verified by treating serum sample with hDDO (inset, green line) or by adding the external standard to serum sample (inset, red line). **b–i** Amount of (**b**) D-aspartate, (**c**) L-aspartate, (**d**) D-aspartate/total aspartate (D + L) ratio, (**e**) L-asparagine; (**f**) D-serine, (**g**) L-serine, (**h**) D-serine/total serine (D + L) ratio and (**i**) glycine in the serum of the patient with *DDO* gene duplication, compared to gender- and age-matched healthy subjects (*n* = 7).

The D-Asp concentration in the patient’s serum was in the same range of control subjects (patient: 0.5035 µM; control: 0.5793 ± 0.038 (mean ± SEM) µM; Fig. 4a, b), while L-Asp levels were ~1.5-fold higher than the mean value of controls (patient: 52.31 µM; control: 29.66 ± 2.04 µM; Fig. 4c). As an index of D-Asp metabolism, we calculated D-Asp/total Asp (D + L enantiomer) ratio, which was ~2-fold lower than in normal subjects (patient: 0.95%; control: 1.98 ± 0.22%; Fig. 4d). We also assessed the patient’s serum concentration of the L-Asp precursor, L-asparagine, which resulted within the range of control group (patient: 94.24 µM; control: 79.64 ± 4.31 µM; Fig. 4e). Finally, we analyzed the serum concentrations of the other D-amino acid present in significant amount in humans, such as D-serine [5–7], its precursor, L-serine [5], as well as glycine. Importantly, both D-serine and glycine are NMDAR co-agonists [5–7]. HPLC analysis revealed that the patient’s levels of these three amino acids, as well as D-serine/total serine ratio, were in the range of control values (D-serine: patient, 4.78 µM—control, 3.84 ± 0.11 µM; L-serine: patient, 354.90 µM—control, 265.40 ± 7.51 µM; D-serine/total serine ratio: patient, 1.33%—control, 1.43 ± 0.05%; glycine: patient, 319.43 µM—control, 280.10 ± 8.47 µM; Fig. 4f–i).

## Discussion

The *DDO* gene encodes for the unique enzyme known to catabolize the endogenous free D-Asp [25, 62, 63], a D-amino acid highly enriched in the brain before birth [15–17, 19–22] and acting as an endogenous agonist of the NMDAR [11, 14, 29] and mGluR5 [12]. Based on this evidence and on postmortem studies indicating lower cortical levels of D-Asp and D-Asp/total Asp ratio in SCZ patients [29, 30], we hypothesized that D-Asp metabolism alteration might affect the normal brain development, which is known to depend on a correct NMDAR and mGluR5 activation [1]. To explore this hypothesis, we used a recently generated *Ddo* knockin mouse model characterized by the insertion of additional *Ddo* gene copy into the genomic locus of *Rosa26*, which produces constitutive *Ddo* overexpression and, consequently, prenatal depletion of cerebral D-Asp [22]. Importantly, the biochemical effect of this genetic manipulation is confined to free cerebral D-Asp metabolism since no alteration in free D-serine or other neuroactive amino acids levels were found in the *Ddo*-overexpressing mouse brain, throughout both prenatal and postnatal life [22]. In the present work, we found that the effect of increased *Ddo* expression on D-Asp metabolism is extended to periphery since *Ddo*-overexpressing mice display also reduced D-Asp levels and D-Asp/total Asp ratio in the blood serum and intestine, compared with wild-type littermates. In line with the critical role of gut microbiota in psychiatric disorders [64, 65] and its ability in synthesizing D-amino acids [66], our evidence let hypothesize a possible contribution of intestine-mediated D-Asp dysmetabolism to the phenotypes observed in *Ddo*-overexpressing mice. Consistent with a possible link between altered D-Asp metabolism and neurodevelopmental alterations, we show that *Ddo*-overexpressing mice present reduced cortical and striatal GMV. This finding is consistent with, and complements earlier observations in humans, where reduced *DDO* gene expression was found to be associated to increased cortical GMV [26]. Moreover, corroborating the relevance of NMDAR stimulation on neuronal proliferation [1] and the influence of D-Asp on adult neurogenesis [27], we found a significant reduction of BrdU-positive cells in the developing cortex of *Ddo*-overexpressing mice, as well as decreased clonal size in neural progenitor cultures overexpressing DDO. Taken together, these results suggest that the reduced number of cortical neurons generated in the dorsal pallium during corticogenesis could lead to reduced cortical GMV in mice with prenatal D-Asp depletion.

In line with a role of cerebral D-Asp metabolism in regulating neurodevelopmental processes, we showed that *Ddo* duplication leads to selective social deficit in female mice at juvenile phase. This evidence arises from the characterization of different domains of social behavior. In particular, in the social interaction test we found that *Ddo*-overexpressing mice showed unaltered reciprocal interaction between pairs, as assessed by evaluating the time spent in sniffing, genital grooming, following and aggression. Similarly, in the sociability phase of the three-chamber test, we revealed unaltered sociability and social motivation (preference for novel mouse vs. inanimate object). Only after the analysis of social novelty phase of the three-chamber test, we unveiled flattened social recognition memory and novelty skills in *Ddo*-overexpressing mice (no discrimination between novel and familiar mouse) [67].

Interestingly, the lack of social recognition memory reported in *Ddo*-overexpressing mice is reminiscent of social behavior deficits documented in animal models with SCZ-like symptoms, associated with neonatal downregulation of the GluN1 subunit of NMDARs [68], or ASD-like phenotypes [39, 69]. Therefore, the present observations support a functional link between precocious D-Asp signaling disruption, early disturbances of NMDAR and neurodevelopmental behavioral abnormalities. However, the unaltered motor stereotypes, social interaction, anxiety and compulsive behaviors found in female *Ddo*-overexpressing mice indicate that metabolic dysfunctions of cerebral D-Asp cannot recapitulate the whole profile of phenotypes observed in animal models of ASD [70, 71]. We hypothesize that gender differences and specificity could have a key role in explaining the absence of broader behavioral psychiatric-like alterations in *Ddo*-overexpressing mice. Indeed, it is well known that while some behavioral deficits described in certain neurodevelopmental disorders are predominantly male‐specific, there are also female‐specific changes that might depend on a complex interaction between genes, sex hormones and environmental factors [72–76]. Therefore, we cannot exclude that the *DDO* gene duplication and the consequent metabolic D-aspartate dysfunction might produce behavioral alterations that depend on the gender, as well as on the age, strain and/or behavioral task used. Further investigations are warranted to clarify this issue.

Altered expression and dysfunctional activity of NMDAR and mGluR5 may represent a core feature in the pathogenesis of psychiatric disorders with neurodevelopmental origin [2, 3, 77–79]. However, no genetic evidence has so far indicated an association between psychiatric syndromes and *DDO* alterations. In this work, we identified for the first time a paternally inherited duplication of chromosome 6, including the entire *DDO* gene, in a young woman with several behavioral abnormalities, including stereotyped behavior and movements, repetitive behavior, emotional dysregulation and intolerance of changes. Overall, these abnormalities were clearly indicative of ID and ASD-associated symptomatology. In addition, the tangential thought and the non-adherence to the context remain in part explained by patient’s cognitive difficulties, although they should be kept under observation as possible predictors of disorders of thought flow. On the other hand, mood alterations (emotional dysregulation) of the patient could be attributable to environmental contingency. Brain MRI did not show pathological signs. However, this analysis was performed at the age of 11, and therefore brain image examinations, including MRI and magnetic resonance spectroscopy, deserve follow-up to evaluate potential alterations in the present cerebral anatomy of the patient.

In line with previous evidence of altered D-Asp metabolism in the postmortem cortex of SCZ subjects [29–31], the distinctive neuropsychiatric profile of the patient is coherent with the possible involvement of the *DDO* gene duplication and, ultimately, with a decrease in cerebral D-Asp content. Although our HPLC analysis did not reveal gross changes on peripheral levels of D-Asp, we found a remarkable reduction of D-Asp/total Asp ratio, which is regarded as an index of D-Asp metabolism, thus confirming the existence of a dysfunctional interconversion between D- and L-Asp enantiomers in the patient, as compared with age- and gender-matched control individuals. Based on these findings, we propose that the *DDO* duplication might have altered D-Asp metabolism during developmental phases and, consequently, affected the patient’s brain glutamatergic signaling, thus contributing to the emergence of the observed psychiatric symptoms. However, given the novelty of this single case report, more studies will be necessary to confirm the direct involvement of *DDO* duplication and relevant D-Asp dysmetabolism in neurodevelopmental psychiatric conditions.

Interestingly, the good socialization and disposition to relational exchanges shown by the patient is in agreement with intact social interaction and sociability skills of female juvenile *Ddo*-overexpressing mice. Unfortunately, besides the Wechsler test, we could not perform quantitative psychiatric assessments of the patient’s psychopathology and social memory abilities. Therefore, future studies will help to clarify whether also the patient displays selective alterations in the domain of social recognition memory. On the other hand, the absence of further behavioral deficits in *Ddo*-overexpressing mice is apparently in contrast with the wide range of dramatic behavioral alterations observed in the patient with the *DDO* duplication. We hypothesize that this discrepancy depends on a selective species-specific impact of early dysfunctional D-Asp metabolism on neurodevelopmental processes. Indeed, the D-Asp/total Asp ratio reaches around 60% in the human prenatal cortex while it never exceeds 18% in mice [21, 22]. Future studies are mandatory to clarify the still unclear influence of D-Asp metabolism in modulating cortical development among different species of mammals.

The duplication observed by a-CGH also involves parts of *METTL24* and *SLC22A16*, two protein-coding genes located at either end of the duplication. In particular, *SLC22A16* encodes for a member of the organic zwitterion transporter protein family, which transports carnitine and anticancer drugs such as bleomycin [80], while *METTL24* encodes for a member of a family of methyltransferase-like proteins still poorly characterized [81]. Interestingly, we proved that the duplication of chromosome 6 is paternally inherited. The presence of the same alteration in the father, which did not show any apparent functional defect until his precocious death (from myocardial infarction), suggests an influence of additional genetic and/or environmental causes for the proband, consistent with the evidence of “multiple genetic hits” and the occurrence of complex genetic, hormonal and environmental interactions in the emergence of neuropsychiatric disorders [72–76, 82]. In this regard, the exome analysis of the patient indicated the presence of seven known potential pathogenic variants in *SLC6A19*, *PRKCQ*, *UCP3*, *ERCC4*, *TYR*, *SLC12A3* and *SERPINA7* genes. However, six of these variants were at the heterozygous status and associated with recessive disorders. The remaining one in the gene *SLC6A19*, the c.1017-4G>A (rs35329108), was in homozygosity (the two parents showed it in heterozygosity) and is potentially associated with iminoglycinuria/hyperglycinuria (autosomal recessive pathologies involving a defective renal tubular reabsorption of glycine, proline and hydroxyproline) when combined with mutations in genes encoding other SLC amino acid transporters [61]. Hence, it is conceivable that this and the other genetic alterations described above have little, if any, relevance for the clinical status of the proband. In this regard, our HPLC analysis in the proband’s serum did not show substantial variations in glycine levels compared with age- and sex-matched controls. Moreover, previous analysis performed at the age of 11 did not evidence aminoaciduria in the patient.

In conclusion, to our knowledge, this is the first case report of a *DDO* gene duplication in a patient with ID, thought disorders and ASD-associated symptomatology. We propose that the *DDO* duplication in humans may interfere with the correct brain development by influencing early NMDAR- and mGluR5-mediated processes. In agreement with this interpretation, we show that early *Ddo* overexpression by depleting the embryonic cerebral pool of D-Asp induces cortical neurogenesis and cortico-striatal GMV abnormalities that, in turn, likely contribute to the emergence of social recognition memory deficit in female juvenile mice. Thus, while further studies are needed to clarify the underlying pathophysiological mechanisms, this work suggests that cerebral D-Asp metabolism alteration contributes to the pathogenesis of neurodevelopmental disorders in mammals.

## Supplementary information

Supplementary Text
