# Supplementary material for: D-aspartate oxidase gene duplication induces social recognition memory deficit in mice and intellectual disabilities in humans
Source: Transl Psychiatry. 2022 Aug 1;12:305. doi: 10.1038/s41398-022-02088-5 (PMC9343392; doi:10.1038/s41398-022-02088-5)
Supplement: Supplementary file 1 — Supplementary Text [file 41398_2022_2088_MOESM1_ESM.docx]

**Supplemental Information** for

***D-aspartate oxidase* gene duplication induces social recognition memory deficit in mice and intellectual disabilities in humans**

Barbara Lombardo^1,2,^*, Marco Pagani^3,^*, Arianna De Rosa^1,^*, Marcella Nunziato^1,2,^*, Sara Migliarini^4,^*, Martina Garofalo^1,5^, Marta Terrile^6^, Valeria D’Argenio^1,7^, Alberto Galbusera^3^, Tommaso Nuzzo^1,5^, Annaluisa Ranieri^1,2^, Andrea Vitale^1,2^, Eleonora Leggiero^1^, Anna Di Maio^1^, Noemi Barsotti^4^, Ugo Borello^4^, Francesco Napolitano^1,8^, Alessandra Mandarino^9^, Marco Carotenuto^9^, Uriel Heresco-Levy^10,11^, Massimo Pasqualetti^4^, Paolo Malatesta^12,13^, Alessandro Gozzi^3^, Francesco Errico^1,14^, Francesco Salvatore^1,15,@^, Lucio Pastore^1,2, @^, Alessandro Usiello^1,5,@^

^1^CEINGE Biotecnologie Avanzate, 80145, Naples, Italy; ^2^Dipartimento di Medicina Molecolare e Biotecnologie Mediche, Università di Napoli Federico II, 80131, Naples, Italy; ^3^Functional Neuroimaging Laboratory, Center for Neuroscience and Cognitive Systems, Istituto Italiano di Tecnologia, 38068, Rovereto, Italy; ^4^Unità di Biologia Cellulare e dello Sviluppo, Dipartimento di Biologia, Università di Pisa, 56126, Pisa, Italy; ^5^Dipartimento di Scienze e Tecnologie Ambientali Biologiche e Farmaceutiche, Università degli Studi della Campania "Luigi Vanvitelli”, Caserta, Italy;

^6^Dipartimento di Oncologia, Biologia e Genetica, Università di Genova, 16132, Genoa, Italy; present address: Novartis Ireland ltd, D04A9N6, Dublin 4, Ireland; ^7^Dipartimento di Promozione delle Scienze Umane e della Qualità della Vita, Università San Raffaele, 00166, Rome, Italy;^8^Department of Veterinary Medicine and Animal Productions, University of Naples Federico II, 80137, Naples, Italy; ^9^Clinic of Child and Adolescent Neuropsychiatry, Department of Mental Health, Physical and Preventive Medicine, University of Campania "Luigi Vanvitelli”, 80100, Naples, Italy; ^10^Research and Psychiatry Departments, Ezrath Nashim-Herzog Memorial Hospital, 9190501, Jerusalem, Israel; ^11^Hadassah Medical School, Hebrew University, 9190501, Jerusalem, Israel; ^12^Dipartimento di Medicina Sperimentale, Università di Genova, 16132, Genoa, Italy; ^13^Ospedale Policlinico San Martino IRCCS, 16132, Genoa, Italy; ^14^Department of Agricultural Sciences, University of Naples Federico II, 80055, Portici, Italy; ^15^Centro Interuniversitario per Malattie Multigeniche e Multifattoriali e loro modelli animali (Federico II, 80131, Naples; Tor Vergata, Rome and “G. D’Annunzio”, Chieti-Pescara).

* These authors contributed equally to this work.

^@^ Corresponding authors

**Supplementary Figures**

**
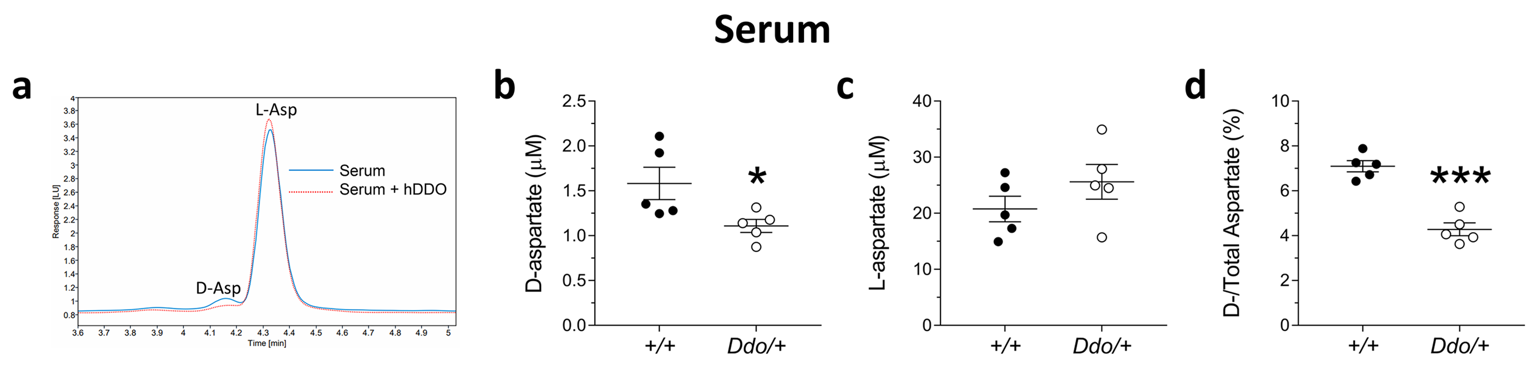
**

**Supplementary Figure 1**. *Determination of D-aspartate and L-aspartate content in blood serum of juvenile R26^Ddo/+^ and R26^+/+^ mice.* (**a**) Overlaid HPLC chromatograms illustrating the specificity of D-aspartate peaks obtained from representative blood serum samples. The identity of the peak corresponding to D-aspartate was verified by treating serum sample with human D-aspartate oxidase (hDDO) (red line). **(b-d)** Amount of **(b)** D-aspartate, **(c)** L-aspartate and **(d)** D-aspartate/total aspartate (D+L) ratio in the serum of *R26^Ddo/+^* mice, compared to *R26^+/+^ mice* (n=5/genotype). Data are expressed as mean ± S.E.M.. **p* < 0.05; ****p* < 0.0001, compared with *R26^+/+^* mice (unpaired two-tailed Student’s *t* test).

**
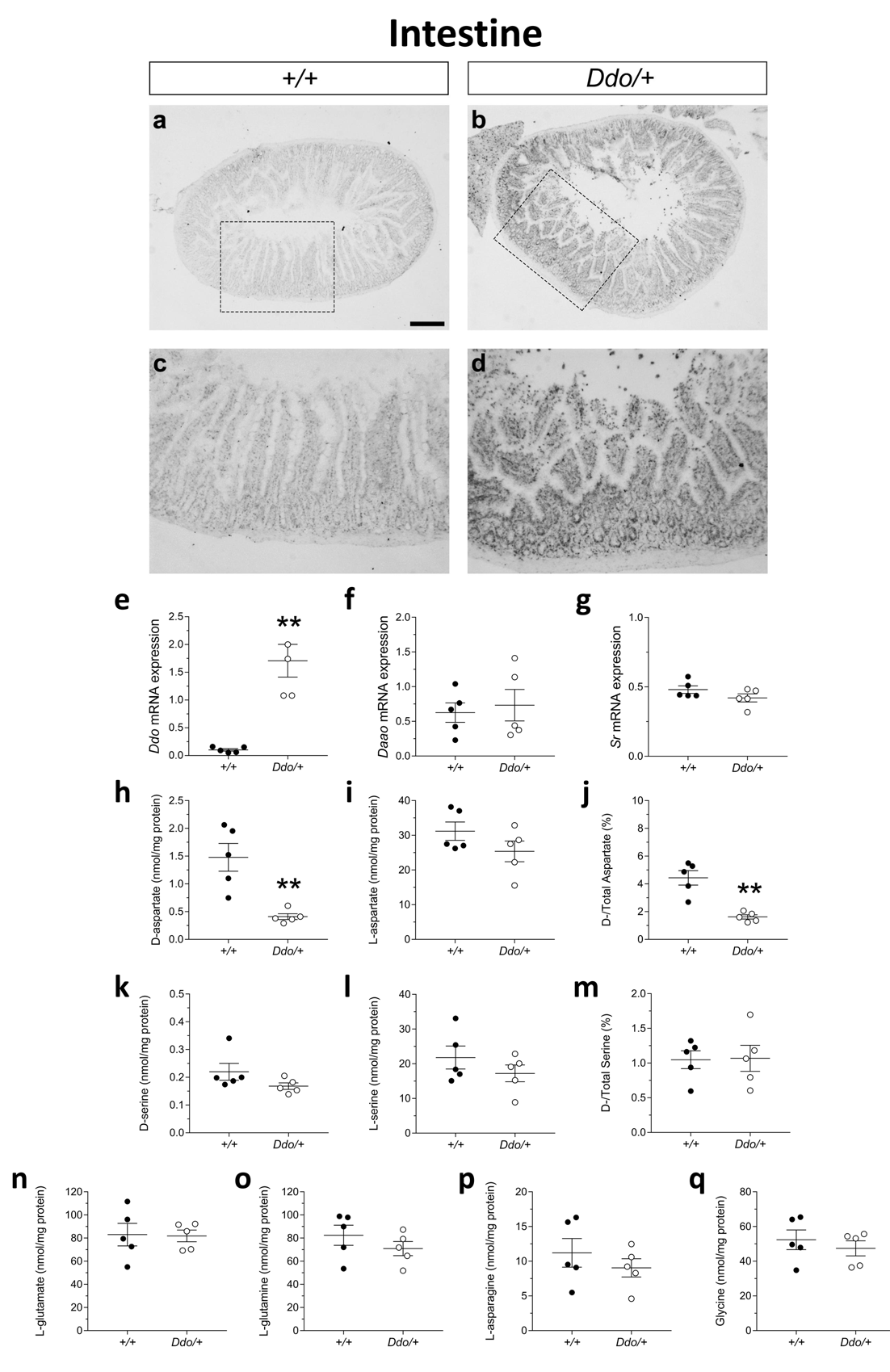
**

**Supplementary Figure 2**. *Determination of D-aspartate metabolism in the intestine of juvenile R26^Ddo/+^ and R26^+/+^ mice.* (**a-d**) Representative images of ileum coronal sections showing *Ddo* expression in **(a,c)** *R26^+/+^* and **(b,d)** *R26^Ddo/+^* juvenile mice. Boxed regions in **(a)** and **(b)** are shown at higher magnification in **(c)** and **(d)**, respectively. **(a,b)** Scale bar 400 mm, **(c,d)** 150 mm. **(e-g)** mRNA expression levels of **(e)** *Ddo*, **(f)** *Daao*, and **(g)** *Sr* genes in the intestine of *R26^Ddo/+^* and *R26^+/+^* juvenile mice (n=5/genotype). **(h-q)** Detection of **(h)** D-aspartate, **(i)** L-aspartate, **(j)** D-aspartate/total aspartate (D+L) ratio, **(k)** D-serine, **(l)** L-serine, **(m)** D-serine/total serine (D+L) ratio, **(n)** L-glutamate, **(o)** L-glutamine, **(p)** L-asparagine and **(q)** glycine in the intestine of *R26^Ddo/+^* mice, compared to *R26^+/+^ mice* (n=5/genotype). Data are expressed as mean ± S.E.M.. ***p* < 0.01, compared with *R26^+/+^* mice (unpaired two-tailed Student’s *t* test).


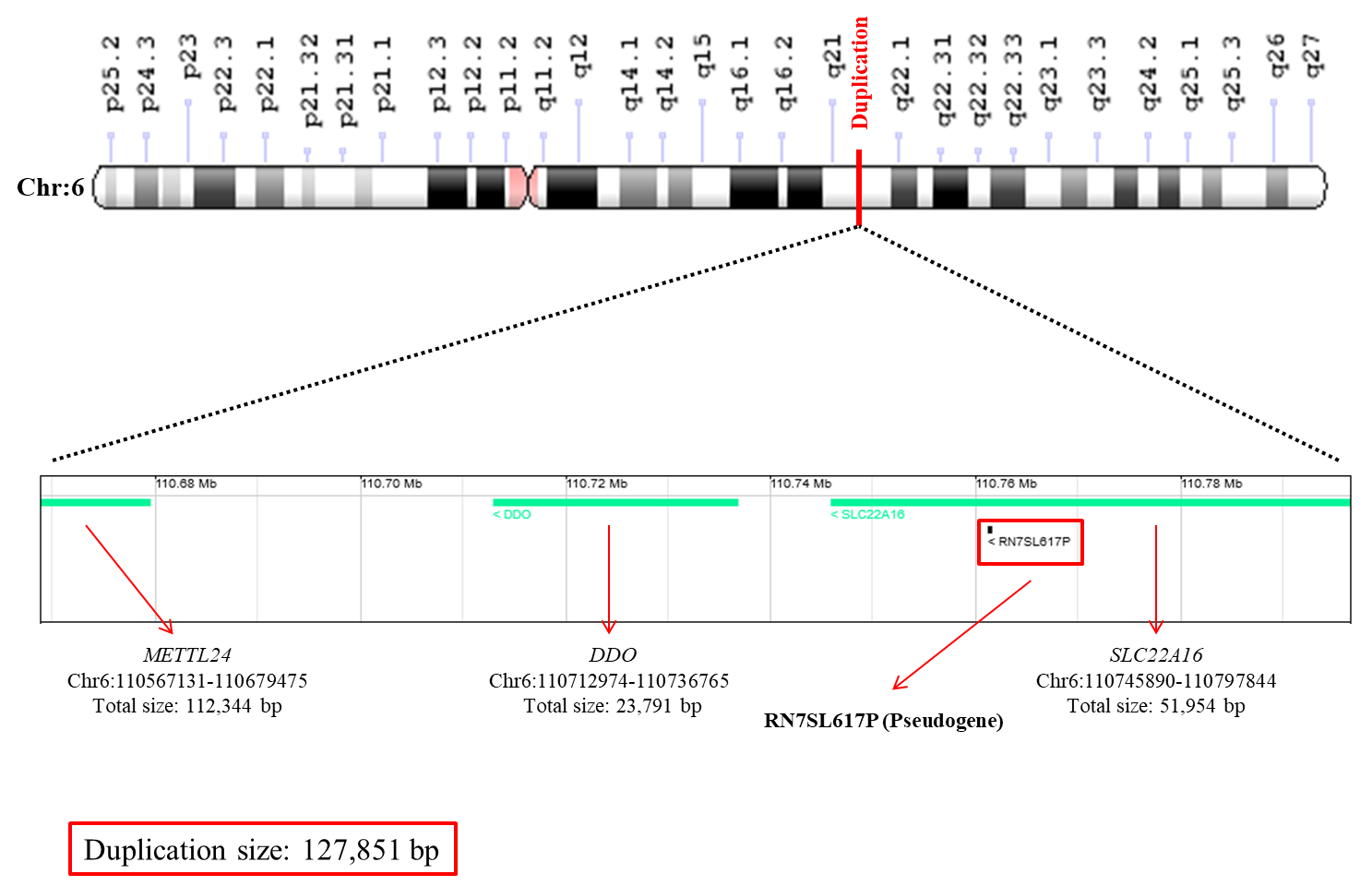


**Supplementary Figure 3.** The duplication observed in the patient on chromosome 6 at q21 region includes the entire *DDO* gene (RefSeq # NC_000006) and part of the methyltransferase like 4 (*METTL24*) gene at the 5’ end of the duplicated DNA segment, and Solute Carrier Family 22 Member 16 (*SLC22A16*) gene at the 3’ end of the same duplication.

**Supplementary Figure 4. Variants' distribution in exome sequencing.** The figure shows the quantity of variants found in the 5 different regions. About 27,000 variants were found in exon regions, about 33,300 in introns, about 5,500 in UTRs and about 3,000 in downstream and upstream regions. Finally,12,000 variants were found in the intergenic regions.

**Supplementary Tables**

**Supplementary Table 1**: Exome Sequencing Raw Data.

| **Patients ID** | **Number of reads** | **Number of reads in bam file passing mapping quality filters** | **Good Quality reads (%)** | **Sequenceable regions with zero coverage (%)** | **Average read depth in analyzable target regions** | **Analyzable target bases with at least 20 reads (%)** | **Number of variants** |
| --- | --- | --- | --- | --- | --- | --- | --- |
| **Proband** | 65,478,691 | 63,122,009 | 96.40% | 2.67% | 89 | 92.57% | 73,734 |
| **Mother** | 63,741,159 | 61,377,827 | 96.30% | 2.61% | 86 | 92.75% | 76,916 |
| **Father** | 65,507,614 | 63,094,702 | 96.30% | 2.53% | 90 | 92.60% | 67,675 |

**Supplementary Table 2.** Quantitative PCR primers used in this study.

| **Primer*** | **Sequence (5’-3’)** | **Gene** | **Product size (bp)** |
| --- | --- | --- | --- |
| **intron 4-5-F** | GACAACATTCAGGAAACTGGGTG | *DDO* | 81 |
| **intron 4-5-R** | TGGCCTCTTGGAAAGAGATCAA |  |  |
|  |  |  |  |
| **znf80-F** | CTGTGACCTGCAGCTCATCCT | *ZNF80* | 120 |
| **znf80-R** | TAAGTTCTCTGACGTTGACTGATGTG |  |  |

F, forward; R, reverse.

**Supplementary Table 3.** List of the primer pairs used for *DDO* sequence analysis (in-home designed).

| **#** | **Exon** | **F (5’-3’)** | | **R (5’-3’)** | **Product size (bp)** | |  |
| --- | --- | --- | --- | --- | --- | --- | --- |
| 1 | EX_1 | TGTGGGCAGCCAAACGAG | | CAGCAACTCTCTCAAAACAGCT | 598 | |  |
| 2 | EX_2 | GCCCATGGAGATCTTGTTGC | | AGCCAGCTGTCTTTTGTCAA | 470 | |  |
| 3 | EX_3 | AGATCCTCTGCCACAATGTG | | CACTGCATGGTCTCTGAGGA | 542 | |  |
| 4 | EX_4 | CAGAAGTTGAGGCTGCAGTG | | GCCCTAGCCATTTGTGAGTG | 499 | |  |
| 5 | EX_5A | CCCCAGTTCCTAGGATGTGA | | GCTATTTTCTGCATCCGGGG | 481 | |  |
| 6 | EX_5B | GGAGACTCAAAGATTTTCCCTGT | | CCGTGCTCAGCTTACATGTT | 600 | |  |
| #, number; F, forward; R, reverse. | | |  | | |  | |

**Supplementary Methods**

**In situ hybridization**

*In situ* RNA hybridizations from brain and intestine tissues obtained, respectively, from post-natal day 0 (P0) and juvenile *Rosa26^+/+^* and *Rosa26^Ddo/+^* (*R26^+/+^* and *R26^Ddo/+^,* respectively**)** mice were performed as previously described ^1^. Fresh brain tissue obtained from post-natal day 0 (P0) wild-type and *R26^Ddo/+^* pups was embedded in Tissue Tek (Sakura), frozen at -80 °C and 14 μm cryostat sections were cut in the sagittal plane. The 1.9 kb antisense cRNA probe against *Ddo* was used ^2^. Images were taken with a MacroFluo microscope (Leica) equipped with DS-SMc digital camera (Nikon).

**Mouse tissue collection and serum extraction**

Intestines and serum were collected from *R26*^+/+^ and *R26*^+/^*^Ddo^* mice (n=5/genotype) at juvenile stage. Serum samples were obtained through retro-orbital blood sampling before euthanizing the animals. After retro-orbital sampling, blood was collected in microtubes and left for 1 h at room temperature and then centrifuged (1500 rpm, 10 min 4 °C) for serum separation. For intestine withdrawal, animals were killed, the intestines were dissected out on an ice-cold surface and luminal content washed out with ice-cold PBS. Tissue samples were pulverized in liquid nitrogen and stored at − 80 °C for subsequent processing. All surgical procedures were performed under anesthesia.

**Quantitative Real Time (qRT)-PCR**

RT-PCR analysis of *Ddo*, *Daao*, *Sr* genes was performed in the intestines of juvenile *R26^+/+^* and *R26^Ddo/+^* mice (n=5/genotype), as previously described ^3^. Experiment was repeated and replicated three times.

**HPLC analyses**

Mouse intestine pulverized samples (n=5/genotype) were homogenized in 1:20 (w/v) 0.2 M TCA, sonicated (3 cycles, 10 s each) and centrifuged at 13,000xg for 20 min. All the precipitated protein pellets from intestine samples were stored at -80 °C for protein quantification. Human and mouse serum samples were mixed in a 1:10 dilution with HPLC-grade methanol (900 µl) and centrifuged at 13,000 x g for 10 min. Supernatants were dried, suspended in 0.2 M TCA and then neutralized with 0.2 M NaOH. Samples were then subjected to pre-column derivatization with o-phthaldialdehyde /N-acetyl-L-cysteine in 50% methanol. Diastereoisomer derivatives were resolved on a ZORBAX Eclipse Plus C8 5-μm reversed-phase column (Agilent, 4.6x250 mm) in isocratic conditions (0.1 M sodium acetate buffer, pH 6.2, 1% tetrahydrofuran, 1 ml/min flow rate). A washing step in 0.1 M sodium acetate buffer, 3% tetrahydrofuran and 47% acetonitrile, was performed after every single run. Identification and quantification of amino acids were based, respectively, on retention time and peak areas, compared with those associated with external standards. The identity of the D-Asp peak was further evaluated by selective degradation catalyzed by a recombinant human DDO (hDDO) ^4,5^. hDDO enzyme (12.5 μg) was added to the samples, incubated at 30 °C for 3 h, and subsequently derivatized. Total protein content of intestine homogenates was determined by Bradford assay method, after re-solubilization of the TCA precipitated protein pellets. The detected amino acids levels in intestine homogenates were normalized by the total protein content and expressed as nmol/mg protein; amino acids concentration in the serum were expressed as µM. Amino acids ratios (D-Asp/total Asp and D-serine/total serine) were expressed as percentage (%). Experiment was repeated and replicated three times.

**Structural magnetic resonance imaging (MRI)**

High-resolution morpho-anatomical images were acquired *ex vivo* in PFA-fixed specimens, a procedure used to obtain high-resolution images with negligible confounding contributions from physiological or motion artefacts ^6^. Brains were imaged inside intact skulls to avoid post-extraction deformations. *R26^+/+^* and *R26^Ddo/+^* mice were deeply anesthetized with intraperitoneal thiopental sodium injection, and their brains were perfused in situ via cardiac perfusion ^7^. The perfusion was performed with PBS followed by 4% PFA (100 ml, Sigma-Aldrich). Both perfusion solutions were added with a gadolinium chelate (ProHance, Bracco, Italy) at a concentration of 10 and 5 mM, respectively, to shorten longitudinal relaxation times. Prior to imaging, samples were blotted and then immersed in plastic tubes filled with a proton-free susceptibility-matching fluid (Fomblin Y, Merck, Germany). High-resolution morpho-anatomical T2-weighted MR imaging of mouse brains was performed using a 72 mm birdcage transmit coil, a custom-built saddle shaped solenoid coil for signal reception. For each session, high-resolution morpho-anatomical images were acquired with the following imaging parameters: FLASH 3D sequence with TR = 18 ms, TE = 10 ms, flip angle = 25, matrix size of 260 x 180 x 180, FOV of 1.82 x 1.26 x 1.26 cm, and voxel size of 70 µm (isotropic).

Inter-group morpho-anatomical differences in local GM volumes were mapped using registration-based Voxel Based Morphometry (VBM) ^6,8-10^. High-resolution morpho-anatomical images were corrected for intensity non-uniformity, skull stripped, and spatially normalized to a study-based template using affine and diffeomorphic registrations. Registered images were segmented to calculate tissue probability maps. The separation of the different tissues was improved by initializing the process with the probability maps of the study-based template previously segmented. The Jacobian determinants of the deformation field were extracted and applied to modulate the GM probability maps calculated during the segmentation. This procedure allowed the analysis of GM probability maps in terms of local volumetric variation instead of tissue density. Brains were also normalized by the total intracranial volume to further eliminate overall brain volume variations and smoothed using a Gaussian kernel with a σ of 3 voxel width.

**BrdU incorporation and immunohistochemical analysis**

Brains were dissected out, fixed overnight in 4% paraformaldehyde at 4°C and cryoprotected in 30% sucrose. Brain tissue was frozen and 30 μm cryo-sections were cut on the coronal plane. For BrdU immunostaining, sections were washed in PBS, incubated in 2 N HCl for 30 min at 37°C and then neutralized with 0.1 M tetrasodium borate buffer pH 8.5 for 10 min at room temperature. Sections were incubated overnight in mouse anti-BrdU antibody (1:400, BD Bioscience, cat. 555627) in PBS with 0.3% Triton X-100 and 10% goat serum at 4°C. The next day sections were washed in PBS and incubated with goat anti mouse Alexa Fluor 594-secondary antibody (Invitrogen Antibodies, cat. R-6393) at 4°C overnight. Next day, sections have been washed with PBS and counter stained with DAPI.

Quantification analyses were performed in blind and sample identity was not revealed until correlations were completed. Analysis was performed throughout the rostro-caudal extension of dorsal pallium, and for each brain (n = 4 *Rosa26^+/+^* and n=5 *Rosa26^Ddo/+^* pups), 5-8 adjacent sections were acquired on a Nikon A1 confocal system, using a 20X plan-apochromat objective. Z series of 21 stacks were acquired at 1024X1024 pixel resolution (pixel size: 0.62 μm), with a z-step of 0.5 μm. Double positive cells for BrdU and DAPI were counted in dorsal pallium and cell density was obtained within region of interest that correspond to the dorso-ventral extension of dorsal pallium cortical plate, that was defined by DAPI staining. Results were expressed as relative percentage ± SEM of BrdU+ cells in *Rosa26^Ddo/+^* pups as compared to controls.

**Primary neuronal cultures and retroviral transduction procedures**

Cells were plated at a density of 2.5x10^5^ cells/cm^2^ onto poly-D-Lysine coated coverslips and transduced by either *Ddo-EGFP* or *EGFP* control retroviral vectors immediately after plating and then grown for 7 days in SATO medium. Cultures were then fixed with 4% PFA in PBS for 15 min for further processing.

The cDNA of *Ddo* ^11^ was inserted into the SalI restriction site of the pCEG retroviral vector, kindly provided by Gordon Fishell (The Skirball Institute of Biomolecular Medicine, New York, USA), containing an internal ribosome entry sequence (IRES) followed by the EGFP coding sequence. Control experiments were performed using either pCEG alone. Primary cultures were infected using a low-titre viral supernatant to obtain a maximum of 30 individual clones per well and allow for a subsequent clonal analysis. Immunocytochemical stainings were performed using the rabbit polyclonal antiserum against GFP (1:500 dilution, Invitrogen, cat. A-6455) subsequently revealed with FITC- conjugated anti-rabbit antibodies (1:50 dilution, Invitrogen, cat. O-11038).

For clonal analyses, mean and standard errors were calculated from the values obtained for the cell population contained in each coverslip. In non-clonal experiments, at least 100 cells per coverslip were analyzed and the mean and standard errors were calculated from different coverslips. “nExp” was adopted to denote the total number of independent experiments.

**Mouse behavior**

*Grooming and rearing*: Grooming and rearing behaviors were coded from 19:00–21:00 h (2 h beginning at the initiation of the dark cycle) using customized freeware software (ODlog). The total amount of time (expressed in s) was determined. Grooming included all sequences of face-wiping, scratching/rubbing of head and ears, and full-body grooming. The observer was blinded to genotype during the videotapes scoring.

*Novelty-induced exploration*: The horizontal motor activity (expressed in cm) was evaluated through a computerized video tracking system (Videotrack, Viewpoint S.A. Champagne au Mont d’Or, France) with time as repeated measure (10-min intervals over a 60-min test session).

*Accelerating rotarod test*: Mice were placed on a rotating drum (3 cm of diameter), and the time that each mouse was able to achieve walking on the top of the rod was measured. Fall latency was recorded automatically when the mouse contacted the plate at the base of the rod, which stopped the session trial. The speed of the rotarod accelerated from 4 to 40 RPM over a 5-min period. Mice were given four consecutive trials over a 3-day lasting test. A minimum of 30 min intertrial rest interval was used to avoid fatigue and exhaustion. Fall latency was analyzed using days as repeated measure.

*Open field test*: Mice were individually placed into the center of a clear Plexiglas (40×40×30 cm), open-field arena (approximately 600 lx lighting), and allowed to explore for 30 min through a computerized video tracking system (Videotrack, Viewpoint S.A. Champagne au Mont d’Or, France). The area adjacent to the wall and the central area of the arena were deﬁned for analysis. The percentage of time spent in the central area was determined with time as repeated measure.

*Elevated plus-maze test*: The experimental apparatus is 55 cm lifted from the floor and consists of two open arms and two closed arms situated opposite each other and separated by a 6 cm squared center platform. Mice were individually placed in the center square and allowed to move freely for 5 min. Test sessions were videotaped and the time spent in each arm was recorded by an experimenter blinded to the genotype. All four paws had to cross the entry of the open, closed arm or the center, to be considered an entry.

*Marble burying test*: The cage contained 20 glass toy marbles (15 mm diameter, assorted colors), placed in five rows of four marbles, on top of the unscented bedding that was 3 cm deep. After test completion, the number of marbles that were buried (more than 50% surface area covered by the bedding) by each mouse was counted by an observer blinded to the genotype. Number of buried marbles was analyzed.

*Prepulse inhibition of the acoustic startle response*: Mice were gently handled 5 min per day for a week before the experiment. A test session began by placing a mouse in the Plexiglas cylinder where it was left undisturbed for 5 min at the background noise level (65 dB). A test session consisted of 7 trial types. One trial type was a 40-ms, 120-dB sound burst used as the startle stimulus. There were six different acoustic prepulse plus acoustic startle stimulus trials. The prepulse sound was presented 100 ms before the startle stimulus. The 20 ms prepulse sounds were 70, 74, 78, 82, 86 or 90 dB. Finally, there were trials where no stimulus was presented in order to measure baseline movements in the cylinder. Six blocks of the eight trial types were presented in a pseudorandom order such that each trial type was presented once within a block of eight trials. The average inter-trial interval was 15 s (ranged from 10 to 20 s). The startle response was recorded for 65 ms (measuring the response every 1 ms) starting with the onset of the startle stimulus. The background noise level in each chamber was 65 dB. PPI was calculated as percentage (%) and used as a dependent variable with prepulse sound levels as repeated measure.

*Social interaction*: Two genetically identical mice of the same gender that had been housed separately were placed together in a cage. Before starting the behavioral tests, each mouse was kept in isolation for 10 days. The cage with bedding material was wrapped with black paper and illuminated weakly from above. All mice were allowed to habituate to a similar cage prior to the test. Mice were placed in opposite corners of the cage, and allowed to move freely for 10 min. The duration of the following types of behavior were analyzed from the video images: sniffing (sniffing several body parts of the other mouse, excluding the anogenital region); genital grooming (sniffing the anogenital region of the other mouse); following (following behind the other mouse while touching the nose to the other’s body); aggressive behavior (biting, lunging, wrestling, and offensive lateral attack). The duration of each behavior (expressed in s) was analyzed by a trained observer blind to the genotype using customized freeware software (ODlog).

*Three-chamber sociability and social novelty test*: The social test apparatus consisted of a transparent acrylic box with removable floor and partitions dividing the box into three chambers. The middle chamber (20 cm × 17.5 cm) was half the width of chamber 1 and chamber 2 (20 cm × 35 cm) with 5 cm openings between each chamber which could be closed or open with a lever operated door. Target mice (stranger 1 and stranger 2) and test animal were habituated to being placed inside wire cages for 3 days (10 min/day) before beginning of testing. The empty wire cages used to contain the stranger mice were cylindrical, 11 cm in height, a bottom diameter of 10.5 cm with the bars spaced 1 cm apart. An inverted transparent cup was placed on the top of the cage to prevent the test mice from climbing on the top of the wire cage. The test was divided in four phases, as follows. 1) Habituation of the test mice to the center chamber: the test animal was introduced to the middle chamber and left to habituate for 10 min. 2) Habituation of the mice to all three chambers: the dividers were then raised, and the test animal was allowed to freely explore all three chambers over a 10 min session (pre-test). 3) Test for sociability: following the pre-test, a novel mouse (stranger 1) was introduced into a wire cage in one of the side-chambers and an empty wire cage was placed on the other side-chamber. The test animal was left to explore all three chambers for 10 min (test) to better acquire the identification cues from novel mouse. Time that test animal spent sniffing the novel mouse and the novel object was used as a specific measure of social investigation. 4) Test preference for social novelty: a new unfamiliar mouse (stranger 2) was inserted in the wire cage previously empty and the test animal was left again to explore for a 10 min session. Preference for social novelty was defined as the time spent sniffing the unfamiliar mouse (stranger 2) compared to the familiar one (stranger 1). Time that test animal spent sniffing the object or strangers (expressed in s) was analyzed by an observer blind to the genotype using customized freeware software (ODlog).

## **Array CGH on humans**

DNA digestion, labeling and hybridization were performed according to the manufacturer’s protocols. DNA specimens were analyzed with the Human Genome CGH Microarray kit 4X180K (Agilent Technologies, Santa Clara, CA), with an average probe spacing of 13 Kb and an average alteration resolution of 25 Kb. Microarrays were analyzed on an Agilent G2600D scanner and image files were quantified using Feature Extraction software (V11.5.1.1, Agilent); data were visualized and analyzed with the Genomic Work Bench Standard Edition (V7.0.4.0, Agilent).

**RT-PCR analysis**

Primer pair specific for *DDO* was designed using Primer Express 2.0 software (Applied Biosystems, Carlsbad, CA) with 20–80% GC content and 62-63°C melting temperature; an amplification the *ZNF80* gene were used to normalize data (Suppl. Table 2). Primer specificity was tested using NCBI’s BLAST software ^12^. The SYBR Green I based RT-PCR assay was performed on a 7900 Fast Real Time PCR apparatus (Applied Biosystems) using the Power SYBR Green PCR Master Mix (Applied Biosystems). The assay was carried out in a 10 µl in duplicates in 96-well optical reaction plates (Applied Biosystems).

***DDO* sequencing analyses in the human trio**

Six primer pairs have been designed to amplify all the *DDO* (Transcript ID ENST00000368924.8; RefSeq NM_003649.3) coding regions, their intronic boundaries and the regulatory regions (Suppl. Table 3). Each amplicon was individually amplified, verified for quality on 2% agarose gel, and purified before sequencing reactions. Direct sequencing was performed with the ABI 3100 capillary sequencer (Applied Biosystems Inc., Foster City, CA, USA), and sequence data analysis was carried out using the SeqMan software (DNASTAR, Inc., Madison, Wisconsin, USA). Next, DNA variants were categorized according to Ensembl (https://www.ensembl.org/index.html), ClinVar (https://www.ncbi.nlm.nih.gov/clinvar/) and dbSNP (https://www.ncbi.nlm.nih.gov/SNP/) databases and the possible impact of specific variations at protein level was predicted using VarSome (https://varsome.com) tool. In addition, sequence variants identified in the analyzed trio were compared to an “in house” database of *DDO* single nucleotide polymorphisms (SNPs) previously identified in both schizophrenia patients and controls.

**Exome Sequencing analysis**

Fifty ng of total gDNA for each sample were enzymatically fragmented and adaptors were added to the ends of the obtained fragments. These pre-captured libraries were amplified following the manufacturer’s instructions (SureSelect Clinical Research Exome V2, Agilent Technologies, Santa Clara, CA). Subsequently, the amplified libraries were hybridized with target-specific probes to capture the genomic regions of interest. Finally, index tags were added by PCR amplification, and quality and quantity of the indexed libraries were assessed using the TapeStation 2200 (Agilent Technologies, Santa Clara, CA). The 3 enriched and indexed libraries were pooled in equimolar amounts and the final pool was sequenced on the NextSeq500 using a Mid Output PE2x150 bp kit (llumina Inc., San Diego, CA, USA).

The sequencing platform generates raw data in fastq files format. Fastq files are univocally assigned to each patient thanks to the index sequence and can be exported to be analyzed by specific bioinformatic software. Data analysis was performed using Agilent's Alissa Portal v5.1.3, a bioinformatic tool that incorporates both a tool for sequencing reads alignment and variants calling, and a tool for clinical interpretation of the big data produced. An adapted pipeline was set up according to the manufacturer's instructions (Agilent Technologies, Santa Clara, CA). All pathogenic and likely pathogenic variants were validated by Sanger sequencing. Specific primers were designed using Primer3 free tool version 4.1.0 (http://primer3.ut.ee/) and verified using NIH Primer-BLAST (<https://www.ncbi.nlm.nih.gov/tools/primer-blast/>).

**References**

1 Pelosi, B., Migliarini, S., Pacini, G., Pratelli, M. & Pasqualetti, M. Generation of Pet1210-Cre transgenic mouse line reveals non-serotonergic expression domains of Pet1 both in CNS and periphery. PLoS One, 2014, 9, e104318.

2 Punzo, D., Errico, F., Cristino, L., Sacchi, S., Keller, S., Belardo, C. *et al.* Age-Related Changes in D-Aspartate Oxidase Promoter Methylation Control Extracellular D-Aspartate Levels and Prevent Precocious Cell Death during Brain Aging. J Neurosci, 2016, 36, 3064-3078.

3 De Rosa, A., Mastrostefano, F., Di Maio, A., Nuzzo, T., Saitoh, Y., Katane, M. *et al.* Prenatal expression of D-aspartate oxidase causes early cerebral D-aspartate depletion and influences brain morphology and cognitive functions at adulthood. Amino Acids, 2020, 52, 597-617.

4 Katane, M., Kanazawa, R., Kobayashi, R., Oishi, M., Nakayama, K., Saitoh, Y. *et al.* Structure-function relationships in human d-aspartate oxidase: characterisation of variants corresponding to known single nucleotide polymorphisms. Biochim Biophys Acta Proteins Proteom, 2017, 1865, 1129-1140.

5 Katane, M., Kuwabara, H., Nakayama, K., Saitoh, Y., Miyamoto, T., Sekine, M. *et al.* Rat d-aspartate oxidase is more similar to the human enzyme than the mouse enzyme. Biochim Biophys Acta Proteins Proteom, 2018, 1866, 806-812.

6 Pagani, M., Damiano, M., Galbusera, A., Tsaftaris, S. A. & Gozzi, A. Semi-automated registration-based anatomical labelling, voxel based morphometry and cortical thickness mapping of the mouse brain. J Neurosci Methods, 2016, 267, 62-73.

7 Dodero, L., Damiano, M., Galbusera, A., Bifone, A., Tsaftsaris, S. A., Scattoni, M. L. *et al.* Neuroimaging evidence of major morpho-anatomical and functional abnormalities in the BTBR T+TF/J mouse model of autism. PloS one, 2013, 8, e76655-e76655.

8 Pagani, M., Bifone, A. & Gozzi, A. Structural covariance networks in the mouse brain. Neuroimage, 2016, 129, 55-63.

9 Pagani, M., Bertero, A., Liska, A., Galbusera, A., Sabbioni, M., Barsotti, N. *et al.* Deletion of Autism Risk Gene Shank3 Disrupts Prefrontal Connectivity. J Neurosci, 2019, 39, 5299-5310.

10 Pucilowska, J., Vithayathil, J., Pagani, M., Kelly, C., Karlo, J. C., Robol, C. *et al.* Pharmacological Inhibition of ERK Signaling Rescues Pathophysiology and Behavioral Phenotype Associated with 16p11.2 Chromosomal Deletion in Mice. J Neurosci, 2018, 38, 6640-6652.

11 Errico, F., Pirro, M. T., Affuso, A., Spinelli, P., De Felice, M., D'Aniello, A. *et al.* A physiological mechanism to regulate D-aspartic acid and NMDA levels in mammals revealed by D-aspartate oxidase deficient mice. Gene, 2006, 374, 50-57.

12 Querques, F., Cantilena, B., Cozzolino, C., Esposito, M. T., Passaro, F., Parisi, S. *et al.* Angiotensin receptor I stimulates osteoprogenitor proliferation through TGFβ-mediated signaling. Journal of Cellular Physiology, 2015, 230, 1466-1474.
